# (3*E*)-4-[(Naphthalen-1-yl)amino]­pent-3-en-2-one hemihydrate

**DOI:** 10.1107/S2414314621009895

**Published:** 2021-09-30

**Authors:** Aboubacar Diop, Tidiane Diop, Cheikh Abdou Khadre Diop, Nikolay Tumanov, Zangrando Ennio, Mamadou Sidibé

**Affiliations:** a Laboratoire de Chimie Minérale et Analytique (LA.CHI.MI.A), Département de Chimie, Faculté des Sciences et Techniques, Université Cheikh Anta Diop, Dakar, Senegal; b Departement de Chimie, Université de Namur ASBL, Rue de Bruxelles 61-5000 Namur, Belgium; c Departement of Chemical and Pharmaceutical Sciences, via Giorgieri, 34127-Trieste, Italy; Zhejiang University (Yuquan Campus), China

**Keywords:** crystal structure, naphthalene, amino­penta­none, *N*-naphthyl­pent-3-en-2-one, hydrogen bonding

## Abstract

The title compound, C_15_H_15_NO·0.5H_2_O, was prepared from α-naphthyl­amine and 2,4-penta­nedione in a 1:1 ratio. An intra­molecular N—H⋯O hydrogen bond in the *N*-naphthyl­pent-3-en-2-one mol­ecule involving the amine and carbonyl groups strengthens the structure. The water mol­ecule interacts with two symmetry-related *N*-naphthyl­pent-3-en-2-one mol­ecules *via*by O—H⋯O hydrogen bonds.

## Abstract 2

In the title compound, the dihedral angle between the naphthalene ring system and the amino­pentenone fragment mean plane (C=C—N—C=O) is 76.91 (19)°. In the crystal, a water mol­ecules are connected with the *N*-naphthyl­pent-3-en-2-one mol­ecules by O—H⋯O-type hydrogen bonds.

## Graphical abstract

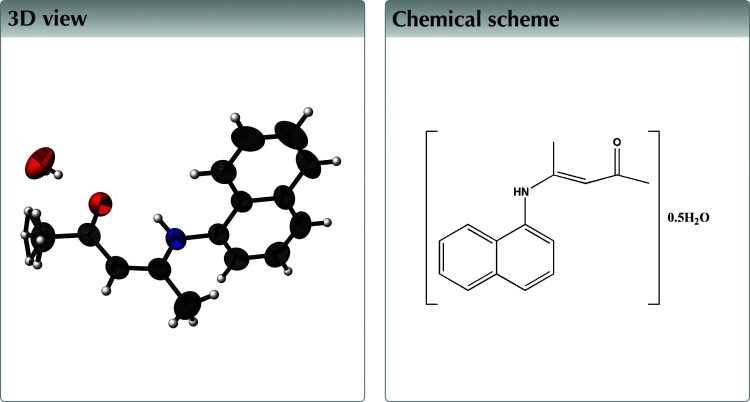

## Structure description

Enamino­nes, which consist of an amino group linked by a carbon–carbon double bond to a carbonyl group, is an area of considerable opportunity (Montanile, 2003 ▸). In fact, enamino­nes are used in the synthesis of different heterocycles and biologically active analogues and also in the development of pharmaceuticals because of their role as organic inter­mediates (Esmaiel *et al.*, 2018 ▸). It should be noted that the biological activity of enamino­ne compounds is attributed to the presence of the active N—C=C—C=O group within a ring system (Kale, 2016 ▸). Much work has been undertaken to explore new routes for the synthesis of enamino­nes. Azzoro *et al.* (1981 ▸) reported a simple method using amines and 1,3-diketones. Eddington *et al.* (2000 ▸) reported on the synthesis and anti­convulsant evaluations of some enamino­nes. In another method, β-chloro vinyl ketone was reacted with amines to synthesize these compounds (Pohland & Benson, 1966 ▸). Other methods of preparation include reactions between formamide dimethyl acetate and ketones (Abdulla & Brinkmeyer, 1979 ▸), acid chlorides with terminal alkynes and tri­ethyl­amine (Karpov & Müller, 2003 ▸), primary amines and β-dicarbonyl compounds catalysed by copper nanoparticles (Kidwai *et al.*, 2009 ▸). Lue & Greenhill (1996 ▸) functionalized enamino­nes by introducing different substituents on the nitro­gen, α- and β-carbon atoms to the carbonyl group. These derivatives are used extensively for preserving natural products and analogues. Enamino­nes are also considered to be good chelating ligands for transition metals in coordination chemistry (Esmaiel *et al.*, 2018 ▸). The anions produced from enaminone offer potential isoelectronic alternatives to cyclo­penta­dienyl-based anions and therefore their transition-metal complexes can act as possible alternative catalysts for olefinic polymerization (Pešková *et al.*, 2006 ▸). Imada *et al.* (1996 ▸) transformed β-amino ketones to enamino­nes using palladium while Tan *et al.* (2008 ▸) reported enaminone applications of rhodium compounds containing bidentate ligand systems.

**Figure 1:**
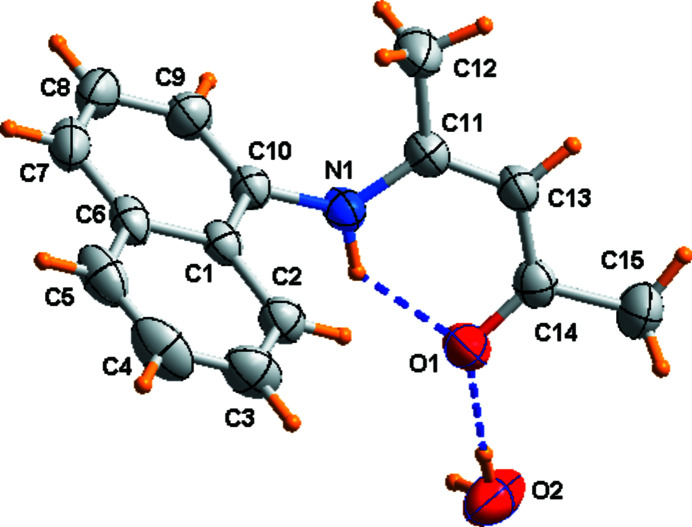
The mol­ecular structure of the title compound with displacement ellipsoids drawn at the 35% probability level. Hydrogen bonds are shown as light-blue dashed lines.

**Figure 2:**
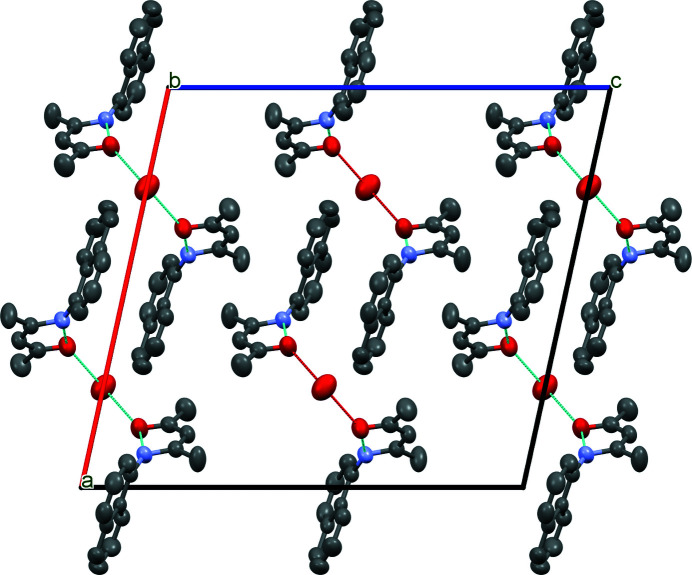
Crystal packing of the title compound viewed down the *b* axis. O—H⋯O hydrogen bonds are shown as light-green dashed lines. H atoms are omitted.

In the title compound (Fig. 1 ▸), the dihedral angle formed by the mean planes through the naphthalene ring system and the amino­penta­none group is 69.66 (9)°. This angle is determined by crystal-packing requirements. The mol­ecular conformation is stabilized by an intra­molecular N—H⋯O hydrogen bond. The water molecule, located on a crystallographic twofold axis, is linked to two symmetry-related *N*-naphthyl­pent-3-en-2-one mol­ecules *via* O—H⋯O hydrogen bonds (Fig. 2 ▸ and Table 1 ▸). In addition, In addition, there are π–π interactions [centroid-to-centroid distance of 3.7975 (10) Å] between the naphthalene ring systems of symmetry-related molecules, generating chains of mol­ecules running in the [100] direction.

## Synthesis and crystallization

A mixture of α-naphthyl­amine (0,143 g; 1 mmol) and 2,4-penta­nedione (0,100 g; 1 mmol) in ethanol solvent was stirred for about 4 h and then filtered. Slow evaporation of the solution at room temperature was carried out, leading to grey crystals suitable for a single-crystal X-ray diffraction study (yield 63%).

## Refinement

Crystal data, data collection and structure refinement details are summarized in Table 2 ▸.

## Supplementary Material

Crystal structure: contains datablock(s) I. DOI: 10.1107/S2414314621009895/xu4045sup1.cif

Structure factors: contains datablock(s) I. DOI: 10.1107/S2414314621009895/xu4045Isup2.hkl

Supporting information file. DOI: 10.1107/S2414314621009895/xu4045Isup3.cml

CCDC reference: 2111316

Additional supporting information:  crystallographic information; 3D view; checkCIF report

## Figures and Tables

**Table 1:** Hydrogen-bond geometry (Å, °)

*D*—H⋯*A*	*D*—H	H⋯*A*	*D*⋯*A*	*D*—H⋯*A*
N1—H1⋯O1	0.905 (17)	1.933 (17)	2.6765 (17)	138.2 (15)
O2—H2*A*⋯O1	0.85 (3)	2.02 (3)	2.8678 (15)	176 (3)

**Table 2:** Experimental details

Crystal data	
Chemical formula	C_15_H_15_NO·0.5H_2_O
*M*_r_	234.29
Crystal system, space group	Monoclinic, *I*2/*a*
Temperature (K)	295
*a*, *b*, *c* (Å)	17.1405 (8), 8.3052 (4), 18.5156 (8)
β (°)	102.347 (4)
*V* (Å^3^)	2574.8 (2)
*Z*	8
Radiation type	Mo *K*α
μ (mm^−1^)	0.08
Crystal size (mm)	0.88 × 0.48 × 0.27

Data collection	
Diffractometer	Oxford Diffraction Xcalibur, Ruby, Gemini Ultra
Absorption correction	Analytical (*CrysAlis PRO*; Rigaku OD, 2018 ▸)
*T*_min_, *T*_max_	0.952, 0.981
No. of measured, independent and observed [*I* > 2σ(*I*)] reflections	6527, 2637, 2045
*R*_int_	0.013
(sin θ/λ)_max_ (Å^−1^)	0.625

Refinement	
*R*[*F* ^2^ > 2σ(*F* ^2^)], *wR*(*F* ^2^), *S*	0.043, 0.123, 1.05
No. of reflections	2637
No. of parameters	168
H-atom treatment	H atoms treated by a mixture of independent and constrained refinement
Δρ_max_, Δρ_min_ (e Å^−3^)	0.13, −0.16

Computer programs: *CrysAlis PRO* (Rigaku OD, 2018 ▸), *SHELXT* (Sheldrick, 2015*a* ▸) and *SHELXL2018/3* (Sheldrick, 2015*b* ▸).

## full crystallographic data

### Crystal data

**Table:**

C_15_H_15_NO·0.5H_2_O	*F*(000) = 1000
*M**_r_* = 234.29	*D*_x_ = 1.209 Mg m^−^^3^
Monoclinic, *I*2/*a*	Mo *K*α radiation, λ = 0.71073 Å
*a* = 17.1405 (8) Å	Cell parameters from 2470 reflections
*b* = 8.3052 (4) Å	θ = 2.9–30.5°
*c* = 18.5156 (8) Å	µ = 0.08 mm^−^^1^
β = 102.347 (4)°	*T* = 295 K
*V* = 2574.8 (2) Å^3^	Block, colourless
*Z* = 8	0.88 × 0.48 × 0.27 mm

### Data collection

**Table:**

Oxford Diffraction Xcalibur, Ruby, Gemini Ultra diffractometer	2637 independent reflections
Graphite monochromator	2045 reflections with *I* > 2σ(*I*)
Detector resolution: 10.3712 pixels mm^-1^	*R*_int_ = 0.013
ω scans	θ_max_ = 26.4°, θ_min_ = 2.3°
Absorption correction: analytical (CrysAlisPro; Rigaku OD, 2018)	*h* = −21→13
*T*_min_ = 0.952, *T*_max_ = 0.981	*k* = −10→10
6527 measured reflections	*l* = −21→23

### Refinement

**Table:**

Refinement on *F*^2^	Primary atom site location: dual
Least-squares matrix: full	Secondary atom site location: dual
*R*[*F*^2^ > 2σ(*F*^2^)] = 0.043	Hydrogen site location: difference Fourier map
*wR*(*F*^2^) = 0.123	H atoms treated by a mixture of independent and constrained refinement
*S* = 1.05	*w* = 1/[σ^2^(*F*_o_^2^) + (0.0578*P*)^2^ + 0.6014*P*] where *P* = (*F*_o_^2^ + 2*F*_c_^2^)/3
2637 reflections	(Δ/σ)_max_ = 0.002
168 parameters	Δρ_max_ = 0.13 e Å^−^^3^
0 restraints	Δρ_min_ = −0.16 e Å^−^^3^

### Special details

**Table:**

Geometry. All esds (except the esd in the dihedral angle between two l.s. planes) are estimated using the full covariance matrix. The cell esds are taken into account individually in the estimation of esds in distances, angles and torsion angles; correlations between esds in cell parameters are only used when they are defined by crystal symmetry. An approximate (isotropic) treatment of cell esds is used for estimating esds involving l.s. planes.

### Fractional atomic coordinates and isotropic or equivalent isotropic displacement parameters (Å^2^)

**Table:**

	*x*	*y*	*z*	*U*_iso_*/*U*_eq_	Occ. (<1)
O1	0.35246 (7)	0.84680 (13)	0.60203 (6)	0.0697 (3)	
N1	0.41810 (7)	0.55405 (15)	0.62641 (6)	0.0554 (3)	
H1	0.4037 (10)	0.641 (2)	0.5971 (9)	0.067*	
C1	0.54448 (8)	0.47503 (16)	0.59592 (7)	0.0487 (3)	
C2	0.57433 (9)	0.63404 (19)	0.60622 (8)	0.0613 (4)	
H2	0.542714	0.714959	0.619655	0.074*	
C3	0.64878 (11)	0.6695 (3)	0.59665 (10)	0.0820 (5)	
H3	0.667331	0.774862	0.602925	0.098*	
C4	0.69744 (11)	0.5507 (3)	0.57766 (10)	0.0914 (6)	
H4	0.748515	0.576743	0.571849	0.110*	
C5	0.67113 (10)	0.3970 (3)	0.56749 (9)	0.0787 (5)	
H5	0.704585	0.318524	0.554932	0.094*	
C6	0.59393 (9)	0.35372 (18)	0.57560 (7)	0.0581 (4)	
C7	0.56410 (11)	0.1952 (2)	0.56424 (9)	0.0716 (5)	
H7	0.596165	0.114794	0.551045	0.086*	
C8	0.48942 (12)	0.15854 (19)	0.57231 (10)	0.0747 (5)	
H8	0.470690	0.053490	0.564663	0.090*	
C9	0.44020 (10)	0.27831 (19)	0.59215 (8)	0.0654 (4)	
H9	0.388734	0.252280	0.596803	0.079*	
C10	0.46696 (8)	0.43196 (16)	0.60462 (7)	0.0511 (3)	
C11	0.40408 (8)	0.57179 (17)	0.69445 (7)	0.0545 (3)	
C12	0.42920 (14)	0.4398 (2)	0.74914 (10)	0.0870 (6)	
H12A	0.395859	0.347308	0.734921	0.131*	
H12B	0.424079	0.475343	0.797244	0.131*	
H12C	0.483825	0.411805	0.750444	0.131*	
C13	0.36721 (9)	0.70740 (18)	0.71367 (8)	0.0583 (4)	
H13	0.357332	0.711308	0.761114	0.070*	
C14	0.34324 (8)	0.84062 (17)	0.66740 (8)	0.0566 (4)	
C15	0.30550 (13)	0.9813 (2)	0.69784 (11)	0.0864 (5)	
H15A	0.297561	1.067498	0.662469	0.130*	0.5
H15B	0.339877	1.017109	0.742911	0.130*	0.5
H15C	0.254936	0.949261	0.707604	0.130*	0.5
H15D	0.297355	0.955081	0.746187	0.130*	0.5
H15E	0.255039	1.005469	0.665745	0.130*	0.5
H15F	0.339980	1.073318	0.701052	0.130*	0.5
O2	0.250000	1.0550 (2)	0.500000	0.1027 (7)	
H2A	0.2805 (17)	0.990 (3)	0.5285 (16)	0.151 (11)*	

### Atomic displacement parameters (Å^2^)

**Table:**

	*U*^11^	*U*^22^	*U*^33^	*U*^12^	*U*^13^	*U*^23^
O1	0.0799 (8)	0.0721 (7)	0.0613 (7)	0.0141 (5)	0.0242 (5)	0.0081 (5)
N1	0.0566 (7)	0.0623 (7)	0.0506 (7)	0.0104 (5)	0.0184 (5)	0.0042 (5)
C1	0.0496 (7)	0.0604 (8)	0.0356 (6)	0.0035 (6)	0.0080 (5)	−0.0002 (6)
C2	0.0641 (9)	0.0675 (9)	0.0528 (8)	−0.0044 (7)	0.0141 (7)	−0.0035 (7)
C3	0.0744 (12)	0.0982 (13)	0.0744 (11)	−0.0283 (10)	0.0180 (9)	−0.0020 (9)
C4	0.0535 (10)	0.148 (2)	0.0751 (12)	−0.0135 (11)	0.0187 (8)	−0.0011 (12)
C5	0.0573 (10)	0.1225 (16)	0.0582 (9)	0.0216 (10)	0.0164 (7)	0.0010 (10)
C6	0.0596 (8)	0.0751 (10)	0.0395 (7)	0.0158 (7)	0.0108 (6)	0.0001 (6)
C7	0.0924 (13)	0.0661 (10)	0.0562 (9)	0.0226 (9)	0.0157 (8)	−0.0059 (7)
C8	0.1019 (14)	0.0539 (9)	0.0677 (10)	−0.0024 (8)	0.0170 (9)	−0.0089 (7)
C9	0.0687 (10)	0.0653 (9)	0.0634 (9)	−0.0095 (7)	0.0166 (7)	−0.0046 (7)
C10	0.0530 (8)	0.0566 (8)	0.0443 (7)	0.0043 (6)	0.0121 (6)	−0.0005 (6)
C11	0.0550 (8)	0.0613 (8)	0.0484 (7)	−0.0003 (6)	0.0137 (6)	−0.0007 (6)
C12	0.1277 (16)	0.0789 (11)	0.0574 (10)	0.0258 (11)	0.0262 (10)	0.0092 (9)
C13	0.0631 (9)	0.0657 (9)	0.0493 (8)	0.0026 (7)	0.0195 (6)	−0.0032 (7)
C14	0.0510 (8)	0.0618 (8)	0.0584 (9)	−0.0019 (6)	0.0151 (6)	−0.0055 (7)
C15	0.1069 (15)	0.0715 (10)	0.0870 (12)	0.0205 (10)	0.0350 (11)	−0.0041 (9)
O2	0.1283 (19)	0.0622 (11)	0.1037 (16)	0.000	−0.0062 (13)	0.000

### Geometric parameters (Å, º)

**Table:**

O1—C14	1.2548 (17)	C8—H8	0.9300
N1—C11	1.3403 (17)	C9—C10	1.359 (2)
N1—C10	1.4271 (17)	C9—H9	0.9300
N1—H1	0.904 (17)	C11—C13	1.375 (2)
C1—C2	1.414 (2)	C11—C12	1.492 (2)
C1—C10	1.4180 (18)	C12—H12A	0.9600
C1—C6	1.4183 (18)	C12—H12B	0.9600
C2—C3	1.358 (2)	C12—H12C	0.9600
C2—H2	0.9300	C13—C14	1.406 (2)
C3—C4	1.385 (3)	C13—H13	0.9300
C3—H3	0.9300	C14—C15	1.503 (2)
C4—C5	1.354 (3)	C15—H15A	0.9600
C4—H4	0.9300	C15—H15B	0.9600
C5—C6	1.410 (2)	C15—H15C	0.9600
C5—H5	0.9300	C15—H15D	0.9600
C6—C7	1.412 (2)	C15—H15E	0.9600
C7—C8	1.355 (2)	C15—H15F	0.9600
C7—H7	0.9300	O2—H2A	0.85 (3)
C8—C9	1.403 (2)	O2—H2A^i^	0.85 (3)
			
C11—N1—C10	125.30 (12)	C13—C11—C12	120.54 (13)
C11—N1—H1	113.3 (10)	C11—C12—H12A	109.5
C10—N1—H1	119.8 (10)	C11—C12—H12B	109.5
C2—C1—C10	122.75 (12)	H12A—C12—H12B	109.5
C2—C1—C6	118.65 (13)	C11—C12—H12C	109.5
C10—C1—C6	118.60 (13)	H12A—C12—H12C	109.5
C3—C2—C1	120.52 (15)	H12B—C12—H12C	109.5
C3—C2—H2	119.7	C11—C13—C14	125.27 (13)
C1—C2—H2	119.7	C11—C13—H13	117.4
C2—C3—C4	120.85 (18)	C14—C13—H13	117.4
C2—C3—H3	119.6	O1—C14—C13	122.64 (13)
C4—C3—H3	119.6	O1—C14—C15	118.90 (14)
C5—C4—C3	120.41 (16)	C13—C14—C15	118.46 (13)
C5—C4—H4	119.8	C14—C15—H15A	109.5
C3—C4—H4	119.8	C14—C15—H15B	109.5
C4—C5—C6	121.21 (17)	H15A—C15—H15B	109.5
C4—C5—H5	119.4	C14—C15—H15C	109.5
C6—C5—H5	119.4	H15A—C15—H15C	109.5
C5—C6—C7	122.70 (15)	H15B—C15—H15C	109.5
C5—C6—C1	118.35 (15)	C14—C15—H15D	109.5
C7—C6—C1	118.95 (14)	H15A—C15—H15D	141.1
C8—C7—C6	120.91 (14)	H15B—C15—H15D	56.3
C8—C7—H7	119.5	H15C—C15—H15D	56.3
C6—C7—H7	119.5	C14—C15—H15E	109.5
C7—C8—C9	120.32 (15)	H15A—C15—H15E	56.3
C7—C8—H8	119.8	H15B—C15—H15E	141.1
C9—C8—H8	119.8	H15C—C15—H15E	56.3
C10—C9—C8	120.68 (15)	H15D—C15—H15E	109.5
C10—C9—H9	119.7	C14—C15—H15F	109.5
C8—C9—H9	119.7	H15A—C15—H15F	56.3
C9—C10—C1	120.52 (13)	H15B—C15—H15F	56.3
C9—C10—N1	121.20 (13)	H15C—C15—H15F	141.1
C1—C10—N1	118.27 (12)	H15D—C15—H15F	109.5
N1—C11—C13	121.20 (13)	H15E—C15—H15F	109.5
N1—C11—C12	118.26 (13)	H2A—O2—H2A^i^	101 (4)
			
C10—C1—C2—C3	−179.47 (14)	C8—C9—C10—C1	1.7 (2)
C6—C1—C2—C3	0.1 (2)	C8—C9—C10—N1	−178.61 (14)
C1—C2—C3—C4	−0.9 (3)	C2—C1—C10—C9	178.20 (13)
C2—C3—C4—C5	0.7 (3)	C6—C1—C10—C9	−1.40 (19)
C3—C4—C5—C6	0.2 (3)	C2—C1—C10—N1	−1.53 (19)
C4—C5—C6—C7	178.95 (16)	C6—C1—C10—N1	178.86 (11)
C4—C5—C6—C1	−1.0 (2)	C11—N1—C10—C9	76.91 (19)
C2—C1—C6—C5	0.79 (19)	C11—N1—C10—C1	−103.36 (16)
C10—C1—C6—C5	−179.59 (12)	C10—N1—C11—C13	169.20 (13)
C2—C1—C6—C7	−179.15 (13)	C10—N1—C11—C12	−11.1 (2)
C10—C1—C6—C7	0.47 (19)	N1—C11—C13—C14	−2.3 (2)
C5—C6—C7—C8	−179.74 (15)	C12—C11—C13—C14	178.05 (16)
C1—C6—C7—C8	0.2 (2)	C11—C13—C14—O1	1.2 (2)
C6—C7—C8—C9	0.0 (3)	C11—C13—C14—C15	−178.45 (16)
C7—C8—C9—C10	−1.0 (3)		

Symmetry code: (i) −*x*+1/2, *y*, −*z*+1.

### Hydrogen-bond geometry (Å, º)

**Table:**

*D*—H···*A*	*D*—H	H···*A*	*D*···*A*	*D*—H···*A*
N1—H1···O1	0.905 (17)	1.933 (17)	2.6765 (17)	138.2 (15)
O2—H2*A*···O1	0.85 (3)	2.02 (3)	2.8678 (15)	176 (3)
